# Study design and protocol for investigating social network patterns in rural and urban schools and households in a coastal setting in Kenya using wearable proximity sensors

**DOI:** 10.12688/wellcomeopenres.15268.2

**Published:** 2019-08-22

**Authors:** Moses Chapa Kiti, Alessia Melegaro, Ciro Cattuto, David James Nokes

**Affiliations:** 1 Epidemiology and Demography Department, KEMRI-Wellcome Trust Research Programme, Kilifi, 80108, Kenya; 2 Department of Social and Political Sciences, Bocconi University, Milan, Italy; 3 Data Science Laboratory, Institute for Scientific Interchange Foundation, Turin, Italy; 4 Zeeman Institute of Systems Biology and Infectious Disease Research, University of Warwick, Coventry, UK; 5 School of Life Sciences, University of Warwick, Coventry, UK

**Keywords:** contact networks, contact patterns, wearable proximity sensors, infectious disease transmission, respiratory infections

## Abstract

**Background:** Social contact patterns shape the transmission of respiratory infections spread via close interactions. There is a paucity of observational data from schools and households, particularly in developing countries. Portable wireless sensors can record unbiased proximity events between individuals facing each other, shedding light on pathways of infection transmission.

**Design and methods:** The aim is to characterize face-to-face contact patterns that may shape the transmission of respiratory infections in schools and households in Kilifi, Kenya. Two schools, one each from a rural and urban area, will be purposively selected. From each school, 350 students will be randomly selected proportional to class size and gender to participate. Nine index students from each school will be randomly selected and followed-up to their households. All index household residents will be recruited into the study. A further 3-5 neighbouring households will also be recruited to give a maximum of 350 participants per household setting. The sample size per site is limited by the number of sensors available for data collection. Each participant will wear a wireless proximity sensor lying on their chest area for 7 consecutive days. Data on proximal dyadic interactions will be collected automatically by the sensors only for participants who are face-to-face. Key characteristics of interest include the distribution of degree and the frequency and duration of contacts and their variation in rural and urban areas. These will be stratified by age, gender, role, and day of the week.

**Expected results:** Resultant data will inform on social contact patterns in rural and urban areas of a previously unstudied population. Ensuing data will be used to parameterize mathematical simulation models of transmission of a range of respiratory viruses, including respiratory syncytial virus, and used to explore the impact of intervention measures such as vaccination and social distancing.

## Introduction

### Background

In infectious disease epidemiology, contact networks consist of individuals (nodes) with connections (edges) between them representing interactions that may lead to infection transmission ^1^. For respiratory and other infections that spread via close contact (such as influenza, severe acute respiratory syndrome (SARS), respiratory syncytial virus (RSV) measles, meningitis, ebola, etc.), social contact networks can be used to highlight potential transmission routes ^2^ and identify targeted intervention strategies through predictive mathematical models. Questionnaire surveys have been conventionally used to collect data on contact patterns ^3– 6^ and networks ^7– 10^, with increasing focus on studies in resource poor settings where disease burden is high ^11– 14^. Despite providing invaluable “who-contacts-whom” data that can be incorporated into models of infection transmission and control ^4, 6, 15, 16^, the questionnaire method suffers several setbacks, key being recall bias and low participation rates ^6, 14, 17^. It has been argued that current transmission dynamic models that omit important factors such as frequency, duration and location of contacts do not adequately capture the heterogeneity of transmission that has direct bearing on intervention measures ^18– 20^. Methods have been advanced to overcome these limitations of diary data, in particular automated data collection methods. These include wireless sensors embedded in portable devices such as mobile phones and customized wearable sensors that use Bluetooth and WiFi ^21, 22^, or low power radio frequencies ^23– 25^, to determine proximity and co-location of users.

### Use of wireless proximity sensors to detect social networks

Proximity sensors (henceforth referred to as “sensors”) using low-powered radio frequencies have been used in ‘closed’ settings such as households ^26, 27^, schools ^28– 30^, hospitals ^25, 31– 34^, work-places ^24, 35^ and conferences ^36^ to characterize close contact social networks. The sensor platform in these studies has been designed to collect proximity data only from individuals facing each other while wearing the sensors, representing conversations or actual physical touch that can lead to infection transmission ( www.sociopatterns.org). The majority of studies using this platform reported a high participation rate (≥75%), suggesting that an unobtrusive way of data collection requiring minimal participant intervention elicits better response rates compared to paper diaries ^14^, especially in settings with a high proportion of illiterate individuals ^26^. Saturation of study populations to define full networks rapidly encounters boundaries due to logistic, time and cost constraints. However, methods have been developed to effectively impute missing data by generating synthetic networks structure given the underlying properties and also the demographic characteristics of the study population ^37^. Most importantly, sensors provide a rich temporal data source, even for partial networks, that can be used to investigate plausible characteristics of infection spread on networks structures weighted by frequency and duration of contacts.

A feasibility study on acceptability and utility of using sensors within five households was conducted in Kilifi over three days of the week ^26^. This pioneer study in Africa revealed three key points, particularly relevant to the design of similar studies in resource poor settings. First, individuals were willing to carry the sensors for extended periods of time because they were unobtrusive and did not require user or investigator intervention to collect data. However, the sensors were considered too big to be used on infants. Second, results suggested children spent more time in contact with other children at the household compared to other age groups, while adults appeared to act as bridges between households. Third, within household temporal contact patterns per day were stable across three days of observation, and contacts between individuals of different households were erratic. Residents aged ≥15 years were under-represented due to being away at school or work. This pilot study recommended elaborate community engagement strategies to ensure wider acceptance of study procedures, and proper training of participants on sensor use and storage (e.g. store them separately when sleeping). Current sensors being deployed in recent studies, such as by Ozella *et al*. ^27^ are smaller, lighter, round in shape and with an on-board flash memory that can store data over longer periods of time. These properties have made the sensors more suitable for use in larger population-based studies, suggesting that they can be deployed in more hard-to-reach populations particularly infants who bear the biggest brunt of respiratory infections.

### Infection transmission in schools and households

Schools and households are locations where a high proportion of a population will spend most of their time and individual interactions are frequent and intense ^4, 6, 11, 13^, potentially leading to a high propensity to spreading of respiratory infections ^38– 40^. These intense interactions are poorly understood, particularly in the very young infants. Studies have revealed the role of older siblings, mothers and other household members on the transmission of respiratory infections to infants. School-going children are notable introducers and transmitters of respiratory infections to same-household members ^41– 43^ and to members of other households ^44^. Infants too spend more time with their mothers compared to other household members ^27, 45, 46^, also suggesting the key role that mothers may have to play in the transmission of infection. In addition, other studies revealed that interactions between infants and non-household members were non-negligible but rarely captured ^45^, suggesting that targeting same-household members only to cocoon infants from infections may have a limited impact on transmission ^45, 46^. Measures such as school closure have been shown to be effective in reducing the magnitude of outbreaks by infections that spread via close contacts (e.g. influenza) ^39, 47^, largely dependent on the transmissibility of the virus and the type of school closure (e.g. one class vs entire school ^42^). As a side effect, school closure results in more age- and location-heterogeneous interactions, particularly with adults at home and with students from other schools ^48^. This increases the potential for transmitting infections to students from other schools and to individuals of working ages, thus suggesting that additional interventions such as vaccinating students from neighbouring schools and adults may need to be considered. Larger studies that may capture contact events within and between households (and school settings) are suggested to provide empirical data that is needed in mathematical models that simulate transmission and assess the impact of various control measures.

### Significance and potential impact of the study

To provide greater insight into social network structures in resource poor settings, we propose to study social contact patterns within schools and households and compare and contrast patterns in the urban and rural setting exhibiting different demographic, economic, and socio-cultural characteristics. This will provide key data for use in transmission dynamic models for common respiratory viral and bacterial infections such as RSV and *S. pneumoniae* that are the leading cause of childhood morbidity and mortality in the SSA setting. We also seek to answer the question how we can optimize study design to capture individual and collective properties of networks that are representative of the community.

## Objectives

The general objective of this work is to utilize radio frequency close-proximity sensors to describe and understand the nature of human networks within a low-resource population that have the potential to transmit respiratory infectious diseases. Specifically:

(i) To collect data on close-proximity interactions in schools and households in one rural and one urban location in Kilifi.
(ii) To characterize the number, duration, and temporal dynamics of social contacts and to define the network’s properties and structure, as well as the underlying determinants within the household and school settings.
(iii) To investigate the potential effect of household and school network structures on the spread of respiratory infections using mathematical models.

## Study design

### Study design and site (geographical)

**Figure 1.**
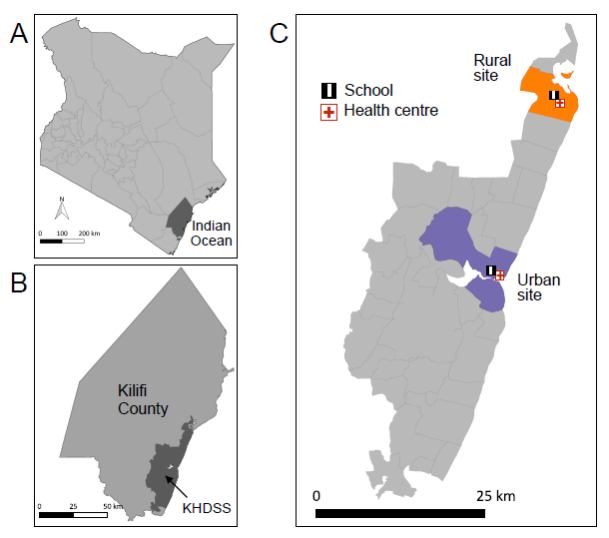
Map showing study locations. ( **A**) The location of Kilifi County within Kenya. ( **B**) The extent of the Kilifi Health and demographic Surveillance Site (KHDSS) within Kilifi county. ( **C**) The locations of rural (orange) and urban (purple) sites, highlighting the location of each of the schools and the nearby health centre.

This is a cross-sectional study conducted in two locations within the Kilifi Health and Demographic Surveillance System (KHDSS) area ^49^, namely Matsangoni and Kilifi Township, categorized as rural and urban, respectively ( Figure 1). These two sites, similar to the other 13 administrative locations covered by KHDSS within Kilifi County, have been under demographic surveillance from April 2002 onwards, and all household demographic information (births, deaths, migrations), and geographic location details have been linked to clinical surveillance data at the Kilifi County Hospital thus creating the KHDSS.

Community social network structure may be affected by structural differences in the social, demographic and economic profiles exhibited in the selected rural and urban locations. For example, rural residents are predominantly subsistence farmers and fishermen, while urban residents are formally employed or are small scale business owners. Households are bigger in the rural, compared to urban, area, with the former having several related families living within the same compound ^49^. School size ranges from 600–900 in Kilifi, with average class sizes being larger and students being slightly older in the rural compared to urban areas. Box 1 contains definitions of terms used.

**Box 1. Definition of terms**

Household. A group of individuals eating from the same kitchen and referring to one of these individuals as the head.

Building unit. A dwelling in which individuals live. One or more building units can form a household.

Index household. The household in which an index student selected from the school resides.

Neighbouring household. One that shares a common fence with the index household (rural), or those that are co-located in a compound owned by one individual (urban).

### Study populations

**Figure 2.**
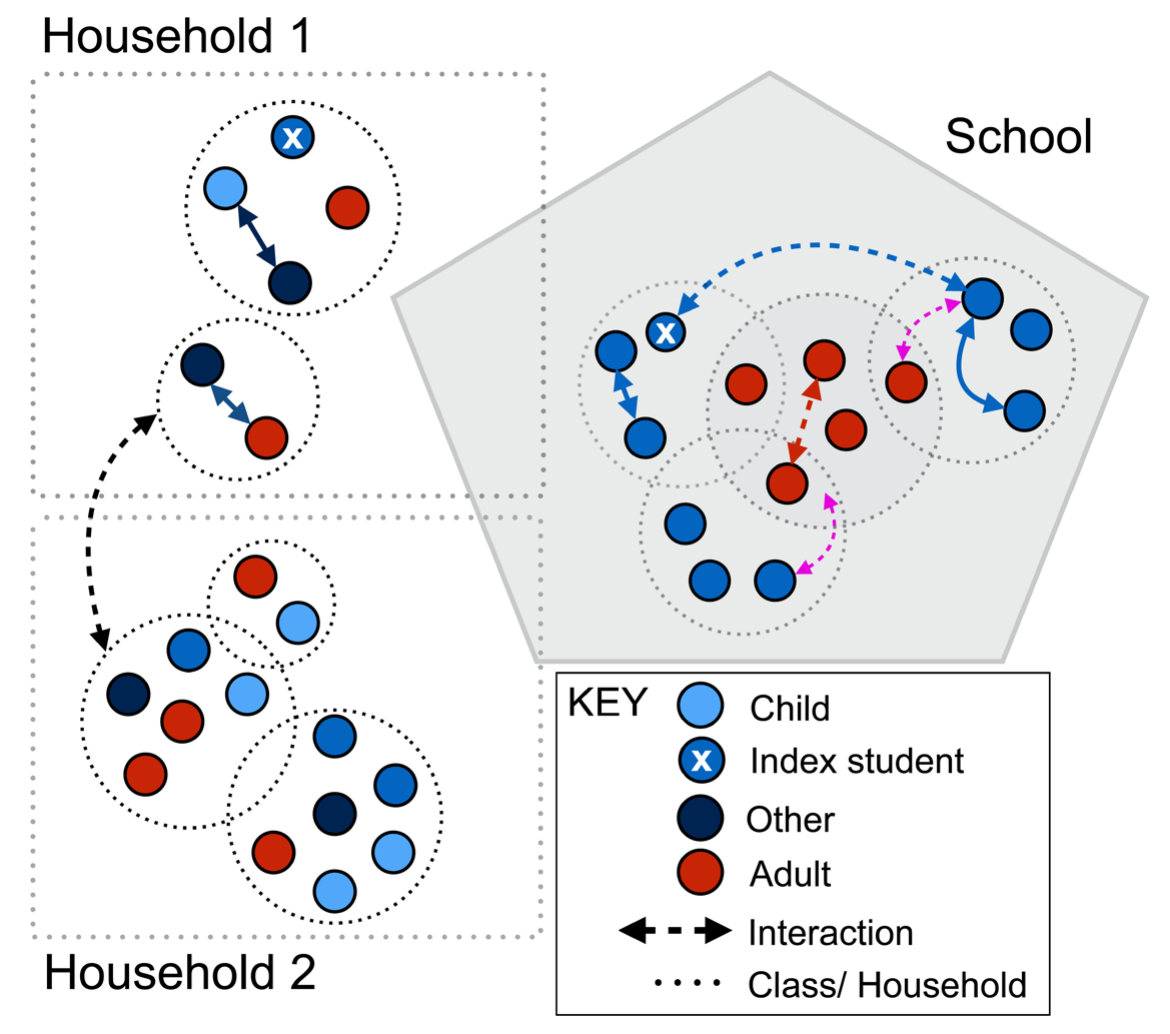
Conceptualization of an interaction framework within and between schools and households. In this scenario, there are children (not attending school), school students and adults. Interactions can occur within or between households, and within and between students and teachers in various classes.

Study recruitment will be done in two phases targeting four sites: rural school and rural households, urban school and urban households. The first point of entry will be two primary schools as identified in Figure 1C. Immediately after collecting data from each school, household residents linked to a sub-selection of participating students will be recruited as depicted in Figure 2. For each site, the maximum number of participants is expected to be 350. This number is guided by the number of sensors available for deployment. Further details are given below.

***School sampling***

Each school will participate if:

(i) The County Education Officer (CEO) gives approval to engage the school. The CEO is responsible for all the administrative matters regarding education in the region. All engagement with the school requires express permission from the officer-in-charge.
(ii) The school includes both primary and early childhood development (ECD) or kindergarten (KG)
(iii) Approval from the Headteacher and school’s Board of Management is given.

Each school will be further stratified into preschool (kindergarten), lower primary (grade 1–4) and upper primary (grade 5–8). Students in these grades are generally within the ages 3–5 years, 6–9 years and 10–15 years, respectively. From the school register, a fixed number (350) of students will be randomly selected proportional to grade and gender. The number of participants per school is limited by the number of sensors available for the experiment. All teachers, approximately 20 per school, will also be recruited into the study.

***Household sampling.*** From each of the defined school stratum (preschool, lower and upper primary), three index students will be selected by simple random sampling. This will give an initial 9 index students per school, who will be linked to their households through data available in the KHDSS. For each of the 9 households per setting, an additional 3–5 (minimum-maximum) neighbouring households will be recruited into the study to give an expected minimum of 396 and 324 residents in rural and urban setting, respectively (assuming average household size is 11 and 9 in rural and urban areas, respectively (unpublished KHDSS data). Individuals within households will be eligible to participate if:

(i) They are a member of a household in which the index student lives, or of a household immediately neighbouring the house of an index student.
(ii) Assent from head of household of an index student, or neighbouring household, is provided.
(iii) The household member provides written consent (teachers, adult, caregiver) or assent (child).

Should more than a third of the expected household members refuse (verbally) to participate, the particular household will be excluded from the study. However, this will not apply if members are away from the household for extended periods due to work or school.

### Study procedures

**Figure 3.**
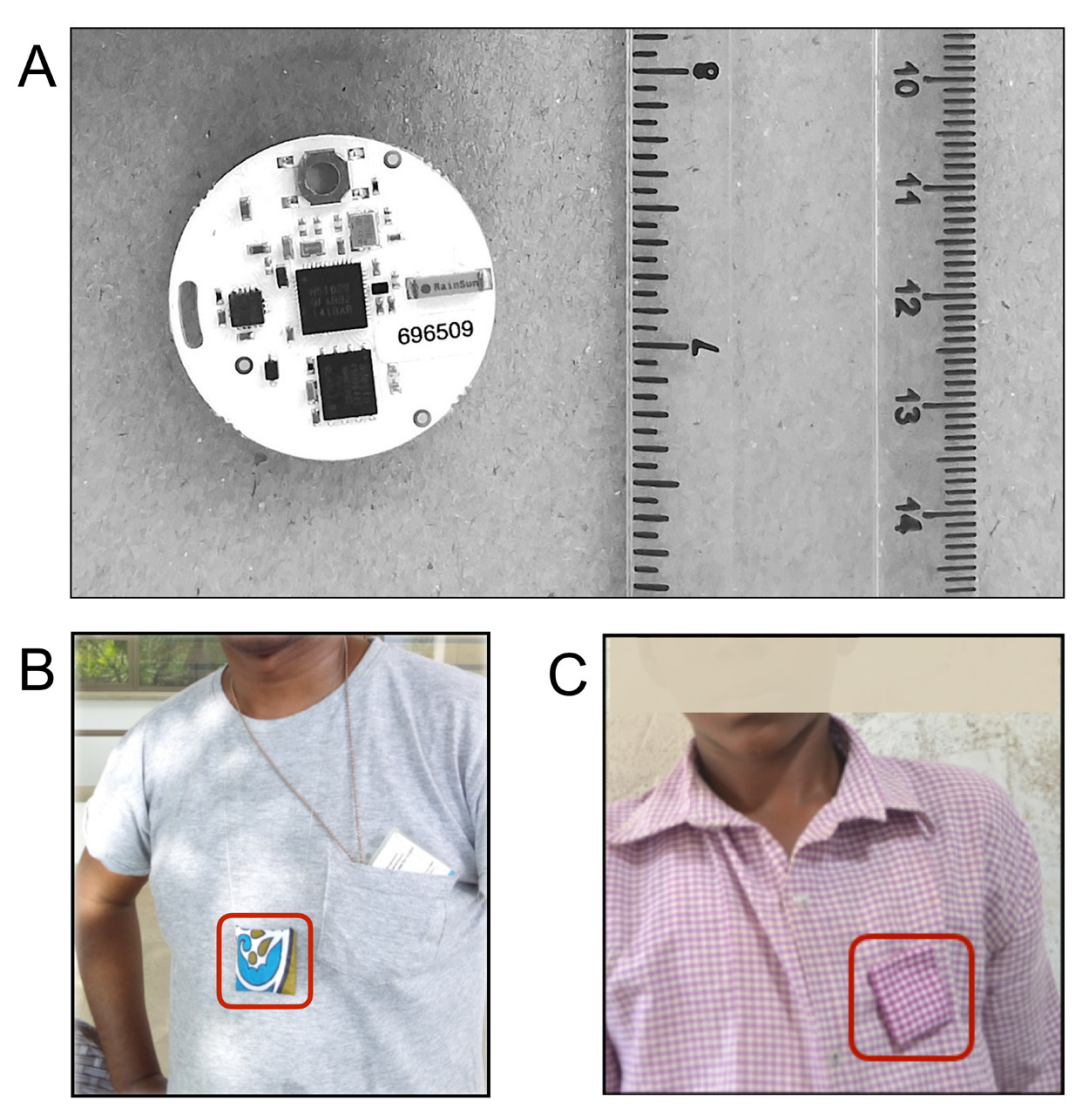
Wireless proximity sensors. ( **A**) A sensor next to a ruler. ( **B**) and ( **C**) How a sensor will be worn by participants, either around the neck with a lanyard or pinned to the shirt pocket, respectively. Household participants will be able to choose the colour of the pouch ( **B**). Students will have a pouch with colour similar to their school shirt/blouse.

***Data collection infrastructure and type.*** Background socio-demographic data for each individual will be extracted from the KHDSS database and updated manually in case some details are missing. Proximity data will be collected using wearable proximity sensors ( Figure 3A), henceforth called sensors. The sensors have been developed by the SocioPatterns project (a European consortium of institutions and investigators focused on social dynamics, www.sociopatterns.org). The sensors operate in the 2.4 GHz ISM band of the RF spectrum. The total weight of the sensor inclusive of a lithium coin battery (CR2032) is <6 grams. Sensors exchange ultra-low-power radio packets in a peer-to-peer fashion by transmitting and scanning their neighbourhood for packets sent by nearby tags on a specific radio channel. Sensors in proximity exchange a maximum of 1 data packet per second and can store over 1,000 hours of continuous data collection in an on-board memory. This exchange of low-power radio packets is used as a proxy for the close co-location of individuals wearing the sensors. The proximity between individuals and temporal resolution are estimated from the power levels and timestamps contained in the data packets, respectively. To estimate how close individuals are, the attenuation of the signals with distance is computed as the difference between the received and transmitted power. Proximity between individuals corresponding to a face-to-face interaction, such as during a handshake, is asserted when the median attenuation over a given time interval exceeds a specified attenuation threshold (in dBm). All individuals will wear a sensor attached to a lanyard around the neck so that it rests on the chest area ( Figure 3B) or pinned to the front of a blouse/shirt especially for younger children ( Figure 3C). In this manner, only face-to-face proximity relations will be detected; moreover, the low-power radio frequency in use cannot propagate through the human body ^24^.

Fieldworkers will ensure participants are properly trained on carrying and storage of devices. During the data collection, some simple measures will be put in place to minimize data loss through not carrying the sensors or deliberately tampering with the sensors. For instance, one class representative will be appointed to ensure that each student participating in the study wears the devices as expected. The head of the household will also be asked to ensure that the correct use of the devices will be maintained throughout the study. This is not expected to be a major role that would have affect the normal class/ household routine.

***Community engagement.*** The following community stakeholders will be consulted prior to field engagement: County Administration and local Chiefs and village elders, County Education Office, KCRs and household heads of identified households. A considerable amount of time may be spent at the school since this will be the first point of entry for the study. Where possible, general consent and assent of all household members of index households may be conducted at the same time if present.

(i) At school: the Headteacher in consultation with the Parent-Teacher Association (PTA) will be asked to give written consent for the school to participate in the study. Once the Headteacher has given approval, the students will be identified through the school register. For easier management, the engagement schedule will be broken down by grade. Parents of selected students will either (a) be requested to come to school for a group consenting exercise, or (b) be followed up to their household for consent by matching household records with those of the students. Only students whose parents give consent will be asked for individual assent to participate. Parents who do not attend the meetings will be traced to their homes by matching their records available in the KHDSS database with the students. Teachers and other staff will also provide individual consent. To minimize disruptions to the normal school routine, engagement sessions with the students will be arranged during their normal breaks, such as class recess, lunchtime or sports breaks. All school engagement will be conducted after obtaining requisite approvals from the relevant County Education Officers.
(ii) At home: approval will be requested from the household head to recruit household members. Other residents will provide individual informed consent (≥ 18 years) or assent (13–17 years) as appropriate. Neighbouring households will be identified during the home visits and consent sought from the household heads. Appointments will be scheduled with individual adult household members.

At the completion of the study, joint feedback meetings with study participants and other stakeholders will be organized per site. At each school on appropriate days, all parents, students and staff will be invited to a health awareness meeting. This will focus on basic hygiene such as hand washing, basic science principles of health research and informing the community on various research activities that KEMRI conducts.

### Data analysis

The sensor firmware and data cleaning procedures have been developed as part of the custom SocioPatterns software developed for this and other studies. The publicly available version of the SocioPatterns tag firmware is a branch of the OpenBeacon firmware that has been developed, tested and verified by the ISI Foundation and the SocioPatterns project. A contact event is detected if one sensor records a radio packet from another sensor, and if the incoming radio power is higher than a given attenuation threshold, calibrated to corresponded to about 1.5 metres of separation distance ^26^. Patterns of contact between participants will be analyzed by statistical distributions describing: a) the number of contacts in households and schools, b) the duration of the contacts, c) the cumulative time spent in contact, and d) the temporal evolution of the networks. Heterogeneity of the contacts and their statistical distributions will be assessed across five key variables: age group (0–4, 5–14, 15–19, 20–49, >50 years), gender (female and male), temporality (hourly, daily and weekly), grade (kindergarten, grades 1–8), and setting (rural/urban). Analysis will be conducted using various Python 2.7 libraries ( Numpy v1.12 and Pandas v0.2) and custom and non-public data processing software by the SocioPatterns project (data cleaning and management), R 3.2.1 (statistical analysis and network visualization), Gephi (network visualization) and QGIS (cartography). Network data analysis and visualization will be aggregated at the school and household level with nodes representing students and household residents, respectively. Links between two individuals *i* and *j* in contact will be weighted by the cumulative duration of interaction between them. Temporal data will be aggregated into time windows of 10 minutes, hourly, daily and over the entire duration of the study (7 days).

**Figure 4.**
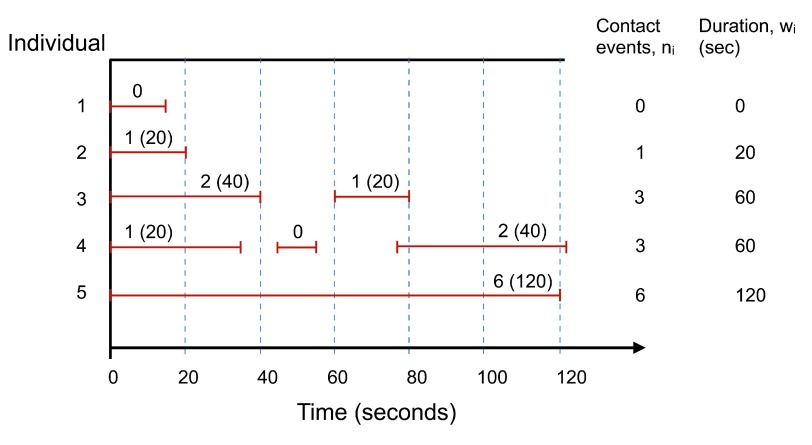
Schema demonstrating the definition of contact event and contact duration. There are 5 individuals. Each red horizontal line represents an interaction between two individuals *i* (1–5) and *j* lasting *s*-seconds. Individual 1 has zero contact events since the duration of contact is <20 seconds. Individual 2 has 1 contact event lasting exactly 20 secs. Individual 3 has three contact events with a 20-sec interruption between the contact events. Individuals 4 and 5 have three and six contact events lasting 60- and 120-seconds, respectively.

***Definition of terms.*** The primary outcome of interest is the median < *k*> degree and corresponding interquartile range (IQR, 25 ^th^ and 75 ^th^ percentiles). For a contact network, the following quantities, similar to a previous household study in Kilifi ^26^, are defined:

(i) A contact event occurs if at least one data packet is exchanged between two devices during a continuous 20-second time window. A contact is considered broken if a 20-second time window passes without data exchange.
(ii) The degree *k*_*i*_ of a node *i* is the number of other nodes to which it was linked during a contact event.
(iii) The weight *n*_*ij*_ of an edge between nodes *i* and *j* is the number of contact events recorded between these individuals during the time window (see Figure 4 for illustration). The mean number of contact events is computed as the sum of the individual contact events divided by the number of nodes, n, $\frac{\sum n_{ij}}{n}.$
(iv) The weight *w*_*ij*_ of an edge between nodes *i* and *j* is the total duration of contact events recorded between these individuals during a given time window (see Figure 4 for illustration). The mean contact duration was computed as the sum of individual contact duration divided by the number of nodes, $\frac{\sum w_{ij}}{n}.$
(v) The network density is the ratio of the number of observed edges formed in a network to the maximum number of expected edges ^27^.
(vi) The clustering coefficient measures the cohesiveness of local groups of nodes by calculating the probability of two different contacts of individual *i* also contacting each other.
(vii) The cosine similarity is defined as an individual’s tendency to have repeated contacts with the same individual over two time points *t*_1_ and *t*_2_, taking into account the duration of contact (weight) *w*_*ij*,1_ and *w*_*ij*,2_ on the edge *i* ↔ *j* measured at time *t*_1_ and *t*_2_^29^, calculated as:
  $$sim(i)=\frac{\sum_{j}\left(w_{ij,1})(w_{ij,2}\right)}{\sqrt{\sum_{j}\left(w_{ij,1}^{2}\right)}\sqrt{\sum_{j}w_{ij,2}^{2}}}\;(1)$$

Essentially, the cosine similarity calculates the changes in the neighbourhood of each node in each pair of daily networks, suggestive of whether a node *i* was in contact with and spent the same amount of time with the same nodes for each successive day pair. Cosine similarity takes values ranging from 0 and 1, with lower values suggesting that neighbouring edges are not the same at time *t*_1_ and *t*_2_, while accounting for time spent in contact. In order to assess the magnitude of the cosine similarities, these values will be compared to a null model. The null model reshuffles the weights of the networks among the edges but does change the topology of the network.

To visualize the networks, the Distributed Recursive Graph Layout (DrL) and Fruchterman-Reingold (F-R) force directed algorithms available in the igraph package in R will be used. In a force-directed algorithm, attractive forces act upon the edges while repulsive forces act between nodes. The F-R algorithm minimizes edge crossing and node overlap thus distributing nodes evenly in the visualization frame while ensuring that the lengths of edges are similar ^50^. Due to this, nodes are clustered together as the density of the links among them increases. The DrL algorithm aims primarily to minimize the overlap of large clusters, or in other words, to emphasize dense clusters ^51^.

## Ethical considerations

### Ethical approval

Ethical approvals were issued by the Scientific and Ethical Review Unit, SERU (KEMRI, Kenya) C/025/3183, and the Biomedical and Scientific Research Ethics Committee, BSREC (University of Warwick, UK) REGO-2016-1738.

### Safety concerns

The sensors and loggers have been used in a previous study in Kilifi that involved piloting the use of sensors in the community, understanding community concerns and learning best practice methods for deployment ^26^, as well as in several other referenced studies. There are no known risks posed by the low power frequency signals emitted by the sensors. The devices will be inserted in a zipped pouch for personal safety, to minimize device loss by theft or misplacement, or data loss through tampering by the participants.

### Informed consent

The informed consent process will be undertaken as described in the community engagement procedures. Consent and assent forms will be back-translated from English to two local languages, Swahili and Giriama. Participants will be free to choose the language in which they would like the information presented. Participants will be free to leave the study or to request the withdrawal of their data at any time and for whatever reason without explanation and without penalty.

### Benefits to participants

Parents who attend the engagement sessions at the school will be refunded travel expenses. We anticipate that this will not exceed USD 2 (~KES 200.00). Participating households will also benefit from health talks that focus on prevention of communicable diseases such as pneumonia and diarrhoea. This will include talks on importance of washings hands (before and after visiting toilets, before handling food, before handling infants, etc), use of handkerchiefs or disposable tissues when sneezing or coughing, and a demonstration of proper hand washing techniques. Each household will then receive two bars of hand-washing soap at the end of data collection.

Schools will benefit from science and health talks from the research team after the data collection. Simple messages focusing on personal hygiene practices that prevent the spread of flu-like infections will be emphasized. Each class will also receive two bars of hand-washing soap. Further, all participating students will receive a stationery pack containing writing materials (writing books, pencils, ruler, mathematical set) and a storybook with information on how to prevent the spreading of flu-like infections.

## Discussion

Respiratory infections and other diseases that are transmitted through close contacts are a predominant cause of morbidity, mortality and healthcare spending in developing countries. Social contact data are important to understand infection patterns since they underpin the transmission dynamics and are key input parameters in mathematical models that evaluate preventative and control measures against these diseases. To date, very few studies have been conducted in sub-Saharan Africa to elucidate the mechanisms of spread. This study proposed to use wireless proximity sensors to collect data from students in schools and household residents in rural and urban areas of coastal Kenya. Schools and households have been identified as the hubs of transmission for respiratory infections such as RSV ^41, 52, 53^ particularly due to prolonged and more intimate contacts at these settings. Results from this study will include the number and duration of contacts and how they vary by age, gender, day of the week, role, and location. With a vaccine against RSV imminent ^54^, predictive modelling can be used to support decision making at the national level for control of infectious disease and important to be based on locally collected data.

There are some limitations to this study. First, it is not possible to describe full social networks at participating school and households due to the limited number of sensors available. Nonetheless, this protocol will investigate contacts within schools and households, settings that are important for spread of respiratory infections. An attempt to minimize selection bias is through random selection of students in a school and linking the students to their entire households and neighbours. This study will also develop the body of knowledge on longitudinal patterns of social networks in rural and urban communities in a developing world setting. Due to the spatially restricted data collection sites, it will not be possible to generalize with certainty these results to other settings locally and globally because of differences in demography, social and cultural attributes. This suggests the need to collect additional national and regional studies, with the advantage that this study protocol can be adapted for use elsewhere since the sensors can be reused. In addition to reusability, the current sensors offer additional advantages when compared to a pilot study conducted in the same setting ^26^: they are smaller and lighter, can be worn by people of all ages including infants, have a bigger memory and battery capacity meaning that data can be collected over longer periods. This may be effectively used to assess the effect of seasonality on changes in social contacts and disease transmission.

## Dissemination

A lay summary of the results will be shared with the participating schools and households, as well as and communities surrounding the schools. Manuscripts will be submitted to appropriate journals discussing the methods, statistical analysis and output, and applications of the data in mathematical modelling of respiratory disease transmission and control.

## Data storage and distribution

Anonymized data will be stored in a repository available online through the KWTRP Research Data Repository on Harvard Dataverse and the SocioPatterns website so as to be findable, accessible, interoperable and reusable (FAIR). Data in de-identified format will be open access to support future use by the wider research community or replication. Access to identifiable data by people outside the investigators and specific collaborators will require permission from the senior investigators, the Data Governance Committee in KEMRI-WTRP, and where necessary, National Ethics Committee. In future, we hope that information collected or generated during this study will be used to support new research by other researchers in Kenya and other countries on other health problems.

## Study status

Data collection started in August 2016 and was completed in April 2017. Currently (20/03/2019), data analysis is ongoing.

## Data availability

No data are associated with this article.
