# Supplementary material for: Study design and protocol for investigating social network patterns in rural and urban schools and households in a coastal setting in Kenya using wearable proximity sensors
Source: Wellcome Open Res. 2019 Aug 22;4:84. Originally published 2019 May 22. [Version 2] doi: 10.12688/wellcomeopenres.15268.2 (PMC6719676; doi:10.12688/wellcomeopenres.15268.2)
Supplement: Peer review history

# Peer review: Reviewer response for version 2

**DOI:** 10.21956/wellcomeopenres.16859.r36298

**Published:** 2019-08-29

**Authors:** Adam J. Kucharski

**Affiliations:** 1 Centre for the Mathematical Modelling of Infectious Diseases (CMMID), London School of Hygiene and Tropical Medicine, London, UK

The authors have addressed my main comments, and hence the article is suitable for approval, subject to one minor final point: I would encourage the authors to consider the logistics in my previous comment 1 (household selection). Their response did not fully address the issue of how they would choose the order in which neighbouring households are selected – presumably it will be random, but worth being clear about this.

I confirm that I have read this submission and believe that I have an appropriate level of expertise to confirm that it is of an acceptable scientific standard.

# Peer review: Reviewer response for version 1

**DOI:** 10.21956/wellcomeopenres.16665.r35915

**Published:** 2019-08-15

**Authors:** Kin-on Kwok

**Affiliations:** 1 The Jockey Club School of Public Health and Primary Care, Stanley Ho Centre for Emerging Infectious Diseases, The Chinese University of Hong Kong, Hong Kong, China

The study design looks quite sound in this protocol. Data generated from this study can be used as the important input in mathematical models to understand transmission dynamics of respiratory diseases in Kenya.

 I had few comments here:

1. Only 9 index cases were randomly recruited in each school in this study according to proportion of gender and grade. In total only 18 kids were recruited from a rural and an urban area in this study while a relative larger number of  teachers (in total 40) were recruited. Is the sample size for the school-aged children large enough to give a good level of resolution of data to reflect kid's contact in school?
2. For the recruitment of household member of each index case, if not all, say more than 2/3 of household members, participated study e.g. mother is not willing to take part of this study. How does the data in the mathematical model reflect the role of those absentees of this study contributing to the disease transmission?
3. I tried to see how number of subjects recruited (396 and 324 residents) can be achieved based on my best understanding. For a recruited school in an urban area, all family members of 9 index cases, 20 teachers and 5 neighbouring households of the index will arrive at 374 residents (assuming 11 households members in each household). Are teachers recruited in this study in the same class as the selected index cases? 
4. It is a cross sectional study. Will there be any other studies in Kenya or neighbouring countries to justify whether there are any seasonal effects on the contact patterns as described in longitudinal contact survey in Hong Kong study ^1^. Will the 7 day period be the same for all individuals? 
5. The author mentioned that this study has an advantage over the diary based data. It is worth to see the discrepancy of between two approaches in this study.
6. Some symbols are not defined (such as n) and consistent with each other (such as ni, nij, wij and wi, # in equation 1, sim (i) should be sim(ij)?).

I confirm that I have read this submission and believe that I have an appropriate level of expertise to confirm that it is of an acceptable scientific standard.

## References

[1] : Temporal variation of human encounters and the number of locations in which they occur: a longitudinal study of Hong Kong residents. *J R Soc Interface*.15(138) : 10.1098/rsif.2017.0838 10.1098/rsif.2017.0838 29367241PMC5805989

# Response

**Published:** 2019-08-19

**Authors:** Moses Kiti

**Affiliations:** KEMRI-Wellcome Trust Research Programme, Kenya

1. Only 9 index cases were randomly recruited in each school in this study according to the proportion of gender and grade. In total, only 18 kids were recruited from a rural and urban area in this study while a relatively larger number of teachers (in total 40) were recruited. Is the sample size for the school-aged children large enough to give a good level of resolution of data to reflect kid's contact in school?

**The actual number of students expected to be recruited randomly per school is 350. This sample size is limited by the number of sensors available for the study, and we expect to get a good resolution of data to reflect the students’ contacts in school. Out of these 350 students, 9 index students will be randomly selected and followed up at their households.**

2. For the recruitment of household member of each index case, if not all, say more than 2/3 of household members, participated study e.g. mother is not willing to take part in this study. How does the data in the mathematical model reflect the role of those absentees of this study contributing to the disease transmission?

**If more than a third of household members are unwilling to participate, that household will not be recruited. However, this household will be replaced by another neighbouring household until the targeted number of households is achieved. In this protocol, we have not provided the full details of the individual-based model. In brief, the model will simulate a population of N-individuals based on the age profile of all residents enumerated in the KHDSS. Each individual will be assigned an expected number of contacts based on the age-specific probability of contact drawn from the data ensuing from this study.**

3. I tried to see how the number of subjects recruited (396 and 324 residents) can be achieved based on my best understanding. For a recruited school in an urban area, all family members of 9 index cases, 20 teachers and 5 neighbouring households of the index will arrive at 374 residents (assuming 11 households members in each household). Are teachers recruited in this study in the same class as the selected index cases? 

From each school, 9 index students will be selected. We will then recruit the entire households of the index students. We will also recruit all residents in 3-5 neighbouring households. Assuming we recruit 3 neighbouring households per index, this will result in {(9*11) + (9*3*11)} = 396 participants for the rural area, as given in the manuscript. The teachers' household members teachers will not be recruited and hence do not account for this number.

4. It is a cross-sectional study. Will there be any other studies in Kenya or neighbouring countries to justify whether there are any seasonal effects on the contact patterns as described in longitudinal contact survey in Hong Kong study ^1^. Will the 7-day period be the same for all individuals? 

**This protocol describes data that will be collected within two sites only (urban and rural) in coastal Kenya. There are no plans to collect data from other regions in Kenya or neighbouring countries in this protocol, but this can be considered for the future. The 7-day period will be the same for all participants in each of the 4 sites (rural school, rural households, urban school, urban households), but data from each site will be collected at separate time-points**.

5. The author mentioned that this study has an advantage over the diary-based data. It is worth to see the discrepancy between two approaches in this study.

**Yes, it is, but we do not plan to collect any diary-based data.**

6. Some symbols are not defined (such as n) and consistent with each other (such as ni, nij, wij and wi, # in equation 1, sim (i) should be sim(ij)?).

***n* refers to the number of sensors, this has now been defined in the protocol. Equation 1 is sim(i) because it refers to the cosine similarity of individual *i*.**

# Peer review: Reviewer response for version 1

**DOI:** 10.21956/wellcomeopenres.16665.r35602

**Published:** 2019-06-07

**Authors:** Adam J. Kucharski

**Affiliations:** 1 Centre for the Mathematical Modelling of Infectious Diseases (CMMID), London School of Hygiene and Tropical Medicine, London, UK

The protocol describes the design of study looking at social interactions in different contexts in Kilifi. It is well motivated, and should generate some nice data relevant to disease transmission.

 I had the following, mostly minor, comments:

- What is the selection process for neighbouring households? For example, if there are 6 neighbouring households, how will only 3-5 be selected? Or if there are only 2 neighbouring households, will a third be chosen? What will happen if some decline to participate?
- Will the 7 day collection period be the same for all individuals in the study? Or will it be site specific?
- Are there plans to collect data on the role within a family (father, mother etc.)? This might be useful for interpreting heterogeneity in contact patterns beyond just age.
- In Figure 2, it wasn't clear to me what the dotted circles represented.
- "the questionnaire method suffers several setbacks, key being recall bias and low participation rates" - there are some studies comparing diaries and sensors (e.g. Mastrandrea *et al.*, 2015 ^1^), so it might be worth considering how the proposed proximity data might relate to existing diary-based studies.

I confirm that I have read this submission and believe that I have an appropriate level of expertise to confirm that it is of an acceptable scientific standard, however I have significant reservations, as outlined above.

## References

[1] : Contact Patterns in a High School: A Comparison between Data Collected Using Wearable Sensors, Contact Diaries and Friendship Surveys. *PLoS One*.2015;10(9) : 10.1371/journal.pone.0136497 e0136497 10.1371/journal.pone.0136497 26325289PMC4556655

# Response

**Published:** 2019-08-19

**Authors:** Moses Kiti

**Affiliations:** KEMRI-Wellcome Trust Research Programme, Kenya

1. What is the selection process for neighbouring households? For example, if there are 6 neighbouring households, how will only 3-5 be selected? Or if there are only 2 neighbouring households, will a third be chosen? What will happen if some decline to participate?

**All households within the Kilifi Health and Demographic Surveillance Site (KHDSS) have been mapped. The selected index households will be highlighted in a map, and the neighbouring households will be identified physically on the ground. Normally, these neighbouring households would share a boundary with the index household. A research assistant will visit the households in succession and recruit a maximum of five. If the head of a household declines participation, then that household will be skipped.**

2. Will the 7-day collection period be the same for all individuals in the study? Or will it be site-specific?

**There are four study sites: rural school, rural households, urban school and urban households. The 7-day period of data collection will be site-specific, and all individuals within the same site will collect data over the same period.** 

3. Are there plans to collect data on the role within a family (father, mother etc.)? This might be useful for interpreting heterogeneity in contact patterns beyond just age.

**Data on family roles will be available from the KHDSS data and will not be collected again.**

4. In Figure 2, it wasn't clear to me what the dotted circles represented. 

**The dotted lines represent a classroom or a household. The figure legend has been updated to include this.**

5. "the questionnaire method suffers several setbacks, the key being recall bias and low participation rates" - there are some studies comparing diaries and sensors (e.g. Mastrandrea *et al.*, 2015), so it might be worth considering how the proposed proximity data might relate to existing diary-based studies.

**We considered this and decided not to collect any paper diary data. We have previously collected data using paper diaries *(Kiti et al., 2014)* and sensors *(Kiti et al., 2016)*, and the decision to collect data using sensors as per this protocol was guided by:**

**1) the ability of individuals to self-record their contacts (two-thirds of participants required shadows in the paper-diary study)**

**2) the overall success of the pilot study using sensors in a rural community.**
